# The impact of young age on cancer-specific and non-cancer-related survival after surgery for colorectal cancer: 10-year follow-up

**DOI:** 10.1038/sj.bjc.6605222

**Published:** 2009-08-11

**Authors:** D C McMillan, C S McArdle

**Affiliations:** 1 Faculty of Medicine, University Department of Surgery, University of Glasgow, Royal Infirmary, Glasgow G31 2ER, UK

**Keywords:** colorectal cancer, age, elective, curative surgery, survival

## Abstract

**Background:**

It has been reported that although young patients present with more advanced disease, when adjusted for stage, cancer-specific survival is not different after surgery for colorectal cancer. However, few studies have examined non-cancer survival in young patients and 10-year survival has rarely been reported. Moreover, the largest study included patients of old age as a comparator. The aim of this study was to compare cancer-specific and non-cancer-related survival at 10 years in a young age cohort and a middle age cohort in patients undergoing surgery for colorectal cancer.

**Methods:**

Two thousand and seventy seven patients who underwent surgery for colorectal cancer between 1991 and 1994 in 11 hospitals in Scotland were included in the study. Ten-year cancer-specific and non-cancer-related survival and the hazard ratios (HR) were calculated according to age groups (<45/45–54/55–64/65–74 years).

**Results:**

On follow-up, 1066 patients died of their cancer and 369 died of non-cancer-related causes. At 10 years, overall survival was 32%, cancer-specific was 45%, and non-cancer-related survival was 72%. On multivariate analysis of all factors, sex (HR 0.77, 95% CI 0.68–0.88, *P*<0.001), mode of presentation (HR 1.64, 95% CI 1.44–1.87, *P*<0.01), Dukes’ stage (HR 2.69, 95% CI 2.49–2.90, *P*<0.001), and specialisation (HR 1.24, 95% CI 1.04–1.44, *P*<0.01) were independently associated with cancer-specific survival. On multivariate analysis of all factors, age (HR 2.46, 2.04–2.97, *P*<0.001), sex (HR 0.56, 0.45–0.70, *P*<0.001), and deprivation (HR 1.16, 1.10–1.24, *P*<0.001) were independently associated with non-cancer-related survival.

**Conclusion:**

The results of this study confirm that young age does not have a negative impact on cancer-specific survival. Moreover, they show that, with 10-year follow-up, young age does not have a negative impact on non-cancer-related survival.

Colorectal cancer is the second commonest cause of cancer death in Western Europe and North America. Many patients have evidence of locally advanced or metastatic disease at the time of initial presentation. Even in those undergoing apparently curative resection, only half survive for 5 years (McArdle and Hole, 2002).

There are a number of factors, in addition to pathological stage, which influence the outcome after surgery for colorectal cancer. These include male gender, deprivation, emergency presentation, and specialisation (McArdle and Hole, 2002).

There is also evidence that old age has a negative impact on 5-year cancer-specific survival after resection for colorectal cancer (Shankar and Taylor, 1998; Colorectal Cancer Collaborative Group, 2000). Furthermore, with 10-year follow-up, the impact of old age appears greater in non-cancer-related compared with cancer-specific survival (McMillan *et al*, 2008).

The impact of young age on cancer-specific and non-cancer-related survival after potentially curative resection is less clear. A number of studies have reported that young patients had similar cancer-specific survival compared with other patients (Bülow, 1980; Järvinen and Turunen, 1984; Isbister and Fraser, 1990; Cusack *et al*, 1996; Chung *et al*, 1998; O’Connell *et al*, 2004; Endreseth *et al*, 2006; Quah *et al*, 2007). However, these studies had limited follow-up and did not report the effect of young age on non-cancer-related survival.

In the largest study to date, O’Connell *et al* (2004) analysed the SEER national cancer database and identified two cohorts based on age. A young cohort consisting of 1334 patients aged between 20 and 40 years was compared with an old cohort consisting of 46 457 patients aged between 60 and 80 years for cancer-specific and non-cancer-related survival. They reported that young patients presented with more advanced disease but had similar cancer-specific survival compared with the old age cohort.

However, old age patients have different cancer-specific and non-cancer-related outcomes compared with the majority of patients, who are middle aged. Indeed, it has been reported that there is an approximate two-fold reduction in cancer-specific survival and a five-fold reduction in non-cancer-related survival in the old age cohort (McMillan *et al*, 2008). Therefore, their previous comparison of young age cohort with an old age cohort may have been inappropriate (O’Connell *et al*, 2004). Moreover, their follow-up was limited to 5 years and they did not examine the effect of young age on non-cancer-related survival in patients undergoing surgery for colorectal cancer.

The aim of this study was to compare cancer-specific and non-cancer-related survival at 10 years in a young age cohort and a middle age cohort in patients undergoing surgery for colorectal cancer.

## Materials and methods

Two thousand and seventy seven patients who underwent a resection for colorectal cancer between 1 January 1991 and 31 December 1994 in 11 hospitals in the central belt of Scotland were included in the study. Information was abstracted from casenotes by two specially trained data managers. Details included age, sex, deprivation category, site of tumour, extent of tumour spread, Dukes’ stage, the nature of surgery, post-operative mortality, and adjuvant therapy. Data for 1991 and 1992 were collected retrospectively, and those for 1993 and 1994 were collected prospectively. There was no difference in baseline characteristics of the patients between the two periods.

The extent of deprivation was defined using the Carstairs Index (Carstairs and Morris, 1991), an area-based measure derived from the 1991 census data based on the postcode of residents at diagnosis. Carstairs divides the scores into a seven-point scale ranging from most affluent (category 1) to most deprived (category 7).

Tumours were classified according to site. The extent of tumour spread was assessed by conventional Dukes’ classification based on histological examination of the resected specimen.

Patients were deemed to have had a curative resection if the surgeon considered that there was no macroscopic residual tumour once resection had been completed.

Individual surgeons were defined as specialists or non-specialists by a panel of six senior consultants and one of the authors (CS McArdle). These assessments were carried out without knowledge of the outcome and before any analysis was performed.

Approval was obtained for information on date and cause of death to be checked with that received by the cancer registration system through linkage with the Registrar General (Scotland). Deaths up to the end of 2003 have been included in the analysis, providing an average length of follow-up of 11 years (minimum 9 years, maximum 13 years).

### Statistical analysis

The percentages of patients surviving 10 years were calculated using the Kaplan–Meier technique. Comparison of the association between age and other variables was carried out using the *χ*^2^-test or a *χ*^2^ for trend where appropriate. The effect of age on cancer- and non-cancer-related survival was examined using Cox's proportional hazards model. Analysis was performed using the SPSS software package (SPSS Inc., Chicago, IL, USA).

## Results

Of the 2077 patients included in the analysis, 3% were aged less than 45 years, 54% were male, 20% were socioeconomically deprived, 63% had colonic tumours, and 49% had Dukes’ A/B disease at the time of surgery. A total of 489 (24%) patients were treated by a specialist surgeon. Four percent of patients received adjuvant therapy.

The baseline characteristics of the patients included in the study are shown in Table 1. With young age there was an increase in the proportion of patients who presented as an emergency (*P*<0.05), who had node-positive disease (*P*<0.01) and died of their cancer. A total of 1066 patients died of their cancer and 369 died of non-cancer-related causes. Of the non-cancer-related deaths, 213 (58%) patients died of cardiovascular or respiratory disease. At 10 years, overall survival was 32%, cancer-specific was 45%, and non-cancer related survival was 72%.

On univariate analysis, sex (*P*<0.001), mode of presentation (*P*<0.001), Dukes’ stage (*P*<0.001), and specialisation (*P*<0.01) were significantly associated with cancer-specific survival (Table 2). On multivariate analysis of all factors, sex (hazard ratio (HR) 0.77, 95% CI 0.68–0.88, *P*<0.001), mode of presentation (HR 1.64, 95% CI 1.44–1.87, *P*<0.01), Dukes’ stage (HR 2.69, 95% CI 2.49–2.90, *P*<0.001), and specialisation (HR 1.24, 95% CI 1.04–1.44, *P*<0.01) were independently associated with cancer-specific survival. When age was entered into the multivariate model as a continuous variable, it remained a nonsignificant predictor of cancer-specific survival (*P*=0.275).

On univariate analysis, age (*P*<0.001), sex (*P*<0.001), and deprivation (*P*<0.001) were significantly associated with non-cancer-related survival (Table 3). On multivariate analysis of all factors, age (HR 2.46, 2.04–2.97, *P*<0.001), sex (HR 0.56, 0.45–0.70, *P*<0.001), and deprivation (HR 1.16, 1.10–1.24, *P*<0.001) were independently associated with non-cancer-related survival. When age was entered into the multivariate model as a continuous variable, it remained a significant predictor of non-cancer-related survival (HR 1.09, 95% CI 1.07–1.11, *P*<0.001).

The relationship between age and the HRs and survival rates for cancer- and non-cancer-related survival is shown in Table 4. Compared with patients under the age of 45 years, those patients who were 45 years or over had no significant difference in their HRs for cancer-specific survival. Compared with patients under the age of 45years, those patients who were 45 years or over had a progressive increase in their HRs (decrease in survival rate) for non-cancer-related survival (*P*<0.001).

## Discussion

The results of this study show that, in patients undergoing resection for colorectal cancer, excluding those of 75 years and over and with 10-year follow-up, more young patients presented as an emergency, had metastatic disease and died of their cancer than of non-cancer-related causes. However, when adjusted for mode of presentation and stage, there was no longer a significant relationship between young age and poorer cancer-specific survival. In contrast, young age was associated, independent of the above factors, with a significant reduction in non-cancer-related survival. Therefore, young age does not appear to have detrimental effect on colorectal cancer outcomes.

There may be a danger that, if only patients undergoing curative resection were included in the analysis, the survival rate of young patients may be overestimated. However, in this study approximately 20% of patient had Dukes’ D disease. Therefore, the present results are consistent with previous studies, which examined the effect of young age on cancer-specific survival in patients with colorectal cancer (Bülow, 1980; Järvinen and Turunen, 1984; Isbister and Fraser, 1990; Cusack *et al*, 1996; Chung *et al*, 1998; O’Connell *et al*, 2004; Quah *et al*, 2007).

A limitation of this study is that the data collection was in part retrospective. However, few studies have examined the relationship between young age and non-cancer survival and only one with 10-year follow-up. Leff *et al* (2007) identified 49 patients aged of 40 years or less at St Marks Hospital between 1982 and 1992 and reported that 10-year overall survival rate was 46%. In this study of 99 patients under the age of 45 years, the 10-year overall survival rate was similar at 43%. However, the overall survival rate was dominated by a cancer-specific survival rate of 46% and the non-cancer survival rate was 95% at 10 years.

The results of this study have a number of important implications. First, patients who develop colorectal cancer at a young age are unlikely to require treatment over and above routine care. Second, if cured from their disease, their lifespan, certainly at 10 years, is unlikely to be altered by their diagnosis with colorectal cancer. Also, as young patients are more likely to present as an emergency or with metastatic disease, it may be that there are delays in the treatment of these young patients in the health-care system, as these patients would not be normally considered at risk of colorectal cancer. Therefore, it remains important that young patients presenting with symptoms suggestive of colorectal cancer are investigated thoroughly.

In summary, the results of this study confirm that young age does not have a negative impact on cancer-specific survival. Moreover, they show that, with 10-year follow-up, young age does not have a negative impact on non-cancer-related survival.

## Figures and Tables

**Table 1:** Baseline characteristics and outcome at 10 years of patients undergoing surgery for colorectal cancer by age (*n*=2077)

	**Age (years)**				
	**<45 years,**	**45–54 years,**	**55–64 years,**	**65–74 years,**	
	***n*=99 (3%)**	***n*=223 (11%)**	***n*=662 (32%)**	***n*=1093 (53%)**	***P*-value**
Female	47 (47%)	110 (49%)	304 (46%)	499 (46%)	
Male	52 (53%)	113 (51%)	358 (54%)	594 (54%)	0.779
					
*Depcat score*					
1, 2	11 (11%)	38 (17%)	138 (21%)	179 (16%)	
3, 4, 5	68 (70%)	135 (61%)	403 (61%)	681 (62%)	
6, 7	18 (19%)	49 (22%)	120 (18%)	232 (21%)	0.651
					
*Mode of presentation*					
Elective	66 (67%)	151 (68%)	478 (72%)	809 (74%)	
Emergency	33 (33%)	72 (32%)	184 (28%)	284 (26%)	0.023
					
*Site*					
Right	23 (23%)	54 (24%)	144 (22%)	286 (26%)	
Left	22 (22%)	28 (13%)	95 (14%)	168 (15%)	
Sigmoid	15 (15%)	57 (26%)	157 (24%)	240 (22%)	
Rectum	37 (37%)	81 (36%)	258 (39%)	389 (36%)	0.134
					
*Dukes’ stage*					
A	6 (6%)	12 (5%)	39 (6%)	85 (8%)	
B	30 (30%)	92 (41%)	282 (42%)	480 (44%)	
C	37 (37%)	77 (35%)	197 (30%)	314 (29%)	
D	26 (26%)	42 (19%)	144 (22%)	214 (20%)	0.008
					
*Specialisation*					
Specialist	28 (29%)	44 (20%)	158 (25%)	259 (24%)	
Non-specialist	67 (71%)	173 (80%)	486 (76%)	808 (76%)	0.974
					
*10-year outcome*					
Alive	43 (43%)	98 (44%)	222 (34%)	279 (26%)	
Cancer death	53 (53%)	116 (52%)	363 (55%)	534 (49%)	
Non-cancer death	3 (3%)	9 (4%)	77 (12%)	280 (26%)	<0.001

**Table 2:** The relationship between clinicopathological characteristics and cancer-specific survival in patients undergoing surgery for colorectal cancer: univariate and multivariate analysis

		**Univariate analysis**		**Multivariate analysis**	
	**Patients (*n*=2077)**	**Hazard ratio (95% CI)**	***P*-value**	**Hazard ratio (95% CI)**	***P*-value**
Age (<45/45–54/55–64/65–74 years)	99/223/662/1093	1.00 (0.93–1.07)	0.972		0.258
Sex (female/male)	960/1117	0.77 (0.68–0.87)	<0.001	0.77 (0.68–0.88)	<0.001
Deprivation (1–2/3–5/6–7)^a^	366/1287/419	1.01 (0.78–1.05)	0.512		0.931
Mode of presentation (elective/emergency)	1504/573	1.66 (1.46–1.89)	<0.001	1.64 (1.44–1.87)	<0.001
Site (right/left/sigmoid/rectum)	507/313/469/765	1.01 (0.96–1.05)	0.757		0.227
Dukes’ stage (A/B/C/D)	142/884/625/426	2.68 (2.49–2.90)	<0.001	2.69 (2.49–2.90)	<0.001
Specialisation (yes/no)	489/1534	1.22 (1.05–1.42)	0.009	1.24 (1.04–1.44)	0.005

Abbreviation: CI=confidence interval.

a Individual deprivation categories were used in the statistical analysis.

**Table 3:** The relationship between clinicopathological characteristics and non-cancer-related survival in patients undergoing surgery for colorectal cancer: univariate and multivariate analysis

		**Univariate analysis**		**Multivariate analysis**	
	**Patients (*n*=2077)**	**Hazard ratio (95% CI)**	***P*-value**	**Hazard ratio (95% CI)**	***P*-value**
Age (<45/45–54/55–64/65–74 years)	99/223/662/1093	2.52 (2.09–3.04)	<0.001	2.46 (2.04–2.97)	<0.001
Sex (female/male)	960/1117	0.56 (0.46–0.70)	<0.001	0.56 (0.45–0.70)	<0.001
Deprivation (1–2/3–5/6–7)^a^	366/1287/419	1.16 (1.09–1.23)	<0.001	1.16 (1.10–1.24)	<0.001
Mode of presentation (elective/emergency)	1504/573	1.09 (0.85–1.38)	0.504		0.324
Site (right/left/sigmoid/rectum)	507/313/469/765	1.06 (0.98–1.14)	0.136		0.140
Dukes’ stage (A/B/C/D)	142/884/625/426	1.10 (0.96–1.25)	0.194		0.067
Specialisation (yes/no)	489/1534	0.90 (0.72–1.14)	0.376		0.659

Abbreviation: CI=confidence interval.

a Individual deprivation categories were used in the statistical analysis.

**Table 4:** Hazard ratios for cancer-specific and non-cancer survival at 10 years in patients undergoing surgery for colorectal cancer by age

	**Cancer specific (deaths=1066)**	**Survival rate**		**Non-cancer (deaths=369)**	**Survival rate**	
**Colorectal (*n*=2077)**	**Hazard ratio (95% CI)**	**% (s.e.)**	***P*-value**	**Hazard ratio (95% CI)**	**% (s.e.)**	***P*-value**
*Age (years)*						
<45	1	46 (5)	0.837	1	95 (3)	<0.001
45–54	0.96 (0.70–1.33)	47 (3)	0.823	1.26 (0.34–4.65)	95 (2)	0.730
55–64	1.05 (0.79–1.40)	43 (2)	0.738	4.11 (1.30–13.03)	81 (2)	0.016
65–74	1.00 (0.76–1.33)	45 (2)	0.994	10.16 (3.26–31.69)	60 (2)	<0.001
